# Crystal structure of De­hydro­dieugenol B methyl ether, a neolignan from *Nectandra leucantha* Nees and Mart (Lauraceae)

**DOI:** 10.1107/S2056989018003717

**Published:** 2018-03-09

**Authors:** Simone S. Grecco, Gerold Jerz, Joao Henrique G. Lago, Peter G. Jones

**Affiliations:** a Institute of Food Chemistry, Technical University of Braunschweig, Schleinitzstrasse 20, 38106 Braunschweig, Germany; b Center of Natural Sciences and Humanities, Federal University of ABC, 09210-580, Santo André, Brazil; c Institute of Inorganic and Analytical Chemistry, Technical University of Braunschweig, Hagenring 30, 38106 Braunschweig, Germany

**Keywords:** crystal structure, neolignan, eugenol

## Abstract

In the title compound, C_21_H_24_O_4_ (systematic name: 4,5′-diallyl-2,2′,3′-tri­meth­oxy­diphenyl ether), the aromatic rings lie almost perpendicular to each other [dihedral angle = 85.96 (2)°]. The allyl side chains show similar configurations, with C_ar_—C—C=C (ar = aromatic) torsion angles of −123.62 (12) and −115.54 (12)°. A possible weak intra­molecular C—H⋯O inter­action is observed. In the crystal, mol­ecules are connected by two C—H⋯O hydrogen bonds, forming undulating layers lying parallel to the *bc* plane. Weak C—H⋯π and π–π stacking inter­actions also occur.

## Abstract 2

In the title compound,the aromatic rings lie almost perpendicular to each other and the allyl side chains show similar configurations. In the crystal, mol­ecules are connected by two C—H⋯O hydrogen bonds, forming undulating layers lying parallel to the *bc* plane.

## Chemical context   

*Nectandra leucantha* belongs to the Lauraceae family, which has a worldwide economic importance (Marques, 2001 ▸). Gottlieb (1972 ▸) described the chemosystematics of the Lauraceae family, highlighting the occurrence of alkaloids, aryl­propano­ids, benzoic esthers, flavonoids, benzo­phenones, fatty acids, mono and sesquiterpenes. The *Nectandra* genus accumulates alkaloids and lignoids as major secondary metabolites (Grecco *et al.*, 2016 ▸). Recent studies from our group describe the anti­parasitical (against *Leishmania donovani* and *Trypanosoma cruzi*) and cytotoxic activities of *N. leucantha* and its isolated metabolites. In terms of chemical composition, neolignans and sesquiterpenes were the major compounds from extracts and essential oils, respectively (da Costa-Silva *et al.*, 2015 ▸; Grecco *et al.*, 2015 ▸, 2017 ▸; de Sousa *et al.*, 2017 ▸). These studies allowed the isolation of C—C- and C—O—C-linked neolignans, including the known isomers de­hydro­dieugenol and de­hydro­dieugenol B, and of the novel compound de­hydro­dieugenol B methyl ether, the object of the present study. In order to confirm the constitution of the title compound, its crystal structure was determined and is reported here.
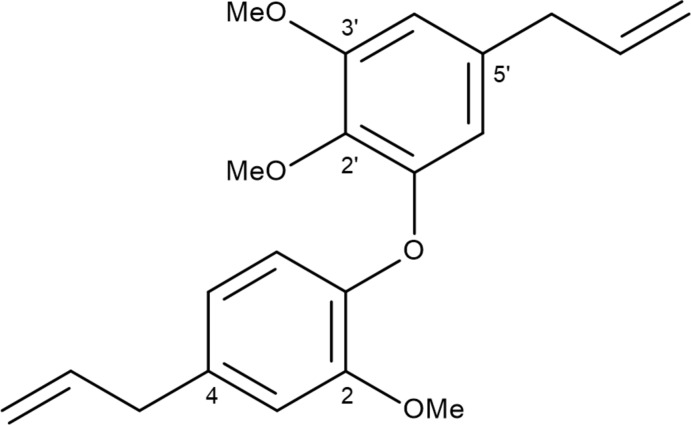


## Structural commentary   

**Figure 1:**
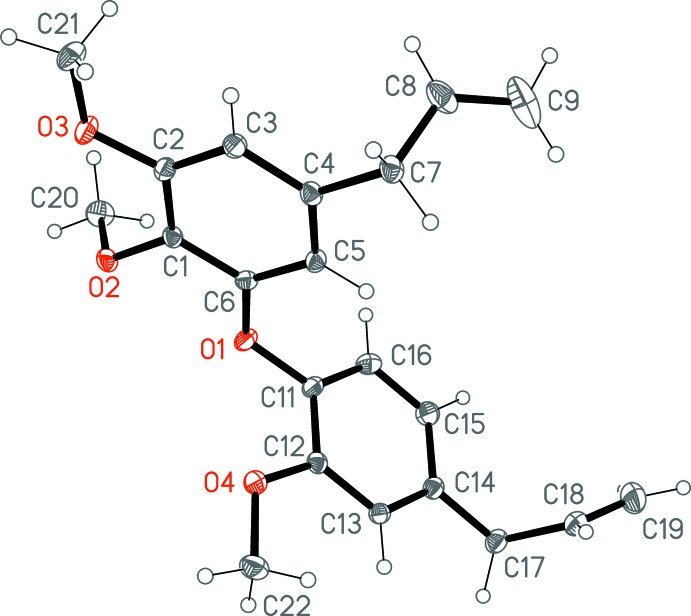
Structure of the title compound in the crystal. Displacement ellipsoids represent 50% probability levels. One hydrogen atom is obscured at each of the atoms C17 and C19.

The mol­ecule of the title compound is shown in Fig. 1 ▸ and selected geometrical data are given in Table 1 ▸. The aromatic rings subtend an inter­planar angle of 85.96 (2)°; the corresponding torsion angles are C1—C6—O1—C11 = −176.28 (8) and C6—O1—C11—C12 = 94.29 (10)°. The allyl side chains show similar configurations, with C4—C7—C8—C9 = −123.62 (12) and C14—C17—C18—C19 = −115.54 (12)°. For the disubstituted (C1–C6) ring, one of the C atoms of the meth­oxy groups (C21) almost lies in the plane of the ring [deviation = 0.064 (1) Å] whereas the other (C20) is significantly displaced [–1.185 (1)Å]. In the other (C11–C16) ring, the meth­oxy carbon atom (C22) lies close to the plane of the ring [deviation = −0.075 (1) Å]. The intra­molecular C20—H20*A*⋯O3 contact with H⋯O = 2.66 Å and an angle of 111°, seems to be at best a borderline inter­action, but it may influence the angle C1—O2—C20, which at 113.39 (7)° is significantly narrower that the other C—O—C angles.

## Supra­molecular features   

**Figure 2:**
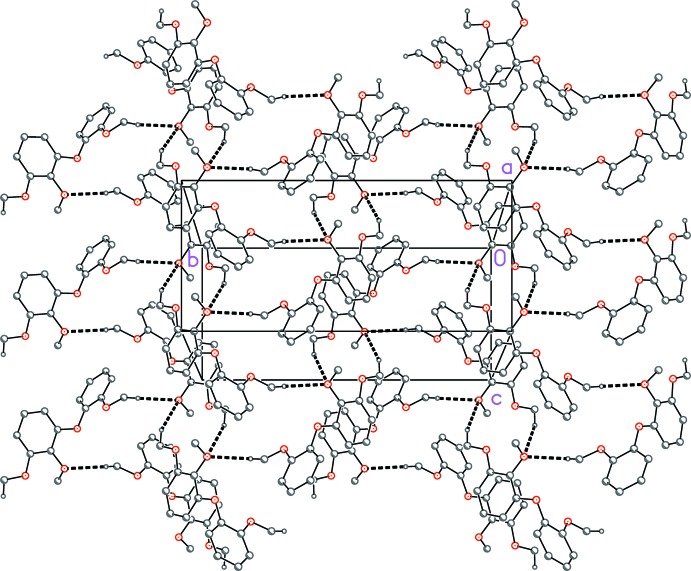
Packing diagram of the title compound viewed perpendicular to the bc plane. For clarity, the allyl side chains and all hydrogen atoms not involved in hydrogen bonding (dashed lines, see Table 2 ▸) have been omitted.

The two weak C—H⋯O hydrogen bonds (Table 2 ▸) link the mol­ecules to form undulating layers parallel to the *bc* plane (Fig. 2 ▸). Additionally, the contacts C19—H19*A*⋯*Cg*(C1–C6) = 2.84 and C17—H17*A*⋯*Cg*(C11-C16) = 2.78 Å (*Cg* = centroid) may represent significant C—H⋯π inter­actions, and the contact of 3.85 Å between centroids of adjacent rings C1–C6 (related by 1 − *x*, 1 − *y*, 1 − *z*) may be a borderline aromatic π–π stacking inter­action.

## Database survey   

The Cambridge Database (Version 5.38; Groom *et al.*, 2016 ▸) contains no examples of 3,4′-di­allyl­diphenyl ethers. Neolignans and related natural products are often isolated as oils, so that crystal structure analyses are rare. In the field of neolignans, lignans, phenyl­propano­ids and eugenyl derivatives the following structures are relevant: Apiculin A and B (BATKAL, BATKEP; Fernandes *et al.*, 2017 ▸); various asarones (AZIQUX01, JAHMUD, JAHNAK, JAHNEO; Qin *et al.*, 2017 ▸); schibitubin A (QANNOL; Liu *et al.*, 2017 ▸); a natural phenyl­propanoid (MIJCAL; Yu *et al.*, 2013 ▸); and several related synthetic compounds (WALSUX, WALTAE, WALTEI, WALTIM; Stomberg *et al.*, 1993 ▸).

## Isolation and crystallization   

*Nectandra leucantha* (Nees & Mart) (Lauraceae) leaves were collected in March 2014, at the Parque Ecologico do Pereque, situated at Cubatão City, State of São Paulo, Brazil. A voucher specimen (EM357) was deposited at the herbarium of the Institute of Biosciences, University of São Paulo, SP, Brazil.

2.5 kg of dried and milled leaves were exhaustively extracted with *n*-hexane, affording 55 g of lipophilic extract after vacuum evaporation of the solvent. In order to increase the content of the neolignan target compounds, the *n*-hexane extract was subjected to a liquid–liquid partition process, using equal parts of *n*-hexane and aceto­nitrile. The neolignan-enrich­ed fraction (NEF – 31.6 g) was obtained from the aceto­nitrile phase after evaporation. A representative amount of 500 mg NEF was subjected to high-performance countercurrent chromatography (HPCCC) fractionation (Ito, 2005 ▸) using a semi-preparative instrument (Spectrum, Dynamic Extractions Ltd, Gwent, UK), a J-type centrifuge equipped with two coil bobbins (PTFE tubing, ID 1.6 mm, column volume 125 ml) operated with the biphasic solvent system *n*-hexa­ne–ethyl acetate–methanol–water (HEMWat 7:3:7:3, *v*/*v*/*v*/*v*) as described by Grecco *et al.* (2017 ▸). The evaluation of biphasic solvent systems was guided by LC–ESI–MS analysis of the respective phase layers to detect a suitable distribution of neolignans. The rotation velocity of the HPCCC centrifuge was set to 1600 rpm (240 G field), and the flow rate of the aqueous mobile phase (5.0 ml min^−1^), and reversed phase operation mode (head-to-tail) resulted in a stationary phase retention of 82.0% after system equilibration. For metabolite profiling and target isolation of neolignans, aliquots of the recovered HPCCC fractions were injected in sequence into an ESI-ion trap MS/MS (HCT Ultra ETD II, Bruker Daltonics, Bremen, Germany) in a standard protocol described by Jerz *et al.* (2014 ▸). This procedure afforded C—C- and C—O—C-linked neolignans, including de­hydro­dieugenol B methyl ether, which was detected in the ESI–MS positive ionization mode with quasimolecular ion signals [*M* + H]^+^ *m*/*z* 341, [*M* + Na]^+^ *m*/*z* 363, and [2*M* + Na]^+^ at *m*/*z* 703 in fractions 51–59 (extrusion mode – volume: 255–295 mL; distribution ratio K_D_: 2.04–2.36). The ESI–MS/MS of *m*/*z* 341 resulted in fragment ions at *m*/*z* and ion intensity [%]: 325.9 (2.3), 299.0 (31.7), 270.9 (34.3), 192.8 (100), 164.8 (52.0), 162.9 (86.9), 149.9 (19.6), 133.0 (47.7) (ESI–MS–parameter: HV capillary – 3500 V, HV end plate offset – 500, dry gas N_2_ 10.0 l min^−1^, nebulizer 60 psi, trap drive 55.6, target mass 500, compound stability 80%, ICC target 100000, ICC on). One-dimensional and two-dimensional NMR data were recorded and compared with those reported previously (Costa-Silva *et al.*, 2015 ▸), confirming the structure as de­hydro­dieugenol B methyl ether. The use of semi-preparative HPCCC, as an all-liquid chromatography technique resulted in a single process step to pure de­hydro­dieugenol B methyl ether. The compound crystallized from the immiscible solvent system by slow evaporation to yield 89 mg. An appropriate colourless block was chosen for X-ray analysis.

## Refinement   

Crystal data, data collection and structure refinement details are summarized in Table 3 ▸. NH hydrogen atoms were refined freely. Methyl hydrogen atoms were refined as idealized rigid groups with C—H 0.98 Å, H—C—H 109.5° (AFIX 137 command). Other hydrogen atoms were included using a riding model starting from calculated positions (C—H_aromatic_ and C—H_vin­yl_ = 0.95, C—H_methyl­ene_ = 0.99, C—H_methine_ = 1.00 Å) with *U*_iso_(H) = 1.2 or 1.5*U*_eq_(C).

## Supplementary Material

Crystal structure: contains datablock(s) I, global. DOI: 10.1107/S2056989018003717/hb7741sup1.cif

Structure factors: contains datablock(s) I. DOI: 10.1107/S2056989018003717/hb7741Isup2.hkl

Supporting information file. DOI: 10.1107/S2056989018003717/hb7741Isup3.cml

CCDC reference: 1827281

Additional supporting information:  crystallographic information; 3D view; checkCIF report

## Figures and Tables

**Table 1:** Selected bond and torsion angles (°)

C6—O1—C11	118.29 (7)	C2—O3—C21	116.94 (7)
C1—O2—C20	113.39 (7)	C12—O4—C22	116.91 (7)
			
C4—C7—C8—C9	−123.62 (12)	C1—C6—O1—C11	−176.28 (8)
C14—C17—C18—C19	−115.54 (12)	C12—C11—O1—C6	94.29 (10)

**Table 2:** Hydrogen-bond geometry (Å, °)

*D*—H⋯*A*	*D*—H	H⋯*A*	*D*⋯*A*	*D*—H⋯*A*
C20—H20*A*⋯O3	0.98	2.66	3.1461 (13)	111
C21—H21*B*⋯O2^i^	0.98	2.54	3.4292 (12)	151
C22—H22*A*⋯O2^ii^	0.98	2.50	3.2885 (12)	138

Symmetry codes: (i) 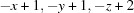; (ii) 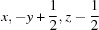.

**Table 3:** Experimental details

Crystal data	
Chemical formula	C_21_H_24_O_4_
*M*_r_	340.40
Crystal system, space group	Monoclinic, *P*2_1_/*c*
Temperature (K)	100
*a*, *b*, *c* (Å)	12.4644 (4), 18.1145 (4), 8.2720 (3)
β (°)	105.835 (3)
*V* (Å^3^)	1796.82 (10)
*Z*	4
Radiation type	Mo *K*α
μ (mm^−1^)	0.09
Crystal size (mm)	0.40 × 0.40 × 0.25

Data collection	
Diffractometer	Oxford Diffraction Xcalibur Eos
No. of measured, independent and observed [*I* > 2σ(*I*)] reflections	46861, 5394, 4749
*R*_int_	0.028
(sin θ/λ)_max_ (Å^−1^)	0.724

Refinement	
*R*[*F* ^2^ > 2σ(*F* ^2^)], *wR*(*F* ^2^), *S*	0.040, 0.102, 1.04
No. of reflections	5394
No. of parameters	229
H-atom treatment	H-atom parameters constrained
Δρ_max_, Δρ_min_ (e Å^−3^)	0.41, −0.24

Computer programs: *CrysAlis PRO* (Agilent, 2014 ▸), *SHELXS97* and *SHELXL97* (Sheldrick, 2008 ▸), *SHELXL2017* (Sheldrick, 2015 ▸) and *XP* (Siemens, 1994 ▸).

## supplementary crystallographic information

### Crystal data

**Table:**

C_21_H_24_O_4_	*F*(000) = 728
*M**_r_* = 340.40	*D*_x_ = 1.258 Mg m^−^^3^
Monoclinic, *P*2_1_/*c*	Mo *K*α radiation, λ = 0.71073 Å
*a* = 12.4644 (4) Å	Cell parameters from 13140 reflections
*b* = 18.1145 (4) Å	θ = 2.8–30.7°
*c* = 8.2720 (3) Å	µ = 0.09 mm^−^^1^
β = 105.835 (3)°	*T* = 100 K
*V* = 1796.82 (10) Å^3^	Block, colourless
*Z* = 4	0.40 × 0.40 × 0.25 mm

### Data collection

**Table:**

Oxford Diffraction Xcalibur Eos diffractometer	4749 reflections with *I* > 2σ(*I*)
Radiation source: Enhance (Mo) X-ray Source	*R*_int_ = 0.028
Detector resolution: 16.1419 pixels mm^-1^	θ_max_ = 31.0°, θ_min_ = 2.3°
ω scan	*h* = −17→17
46861 measured reflections	*k* = −25→25
5394 independent reflections	*l* = −11→11

### Refinement

**Table:**

Refinement on *F*^2^	Primary atom site location: structure-invariant direct methods
Least-squares matrix: full	Secondary atom site location: difference Fourier map
*R*[*F*^2^ > 2σ(*F*^2^)] = 0.040	Hydrogen site location: inferred from neighbouring sites
*wR*(*F*^2^) = 0.102	H-atom parameters constrained
*S* = 1.04	*w* = 1/[σ^2^(*F*_o_^2^) + (0.0455*P*)^2^ + 0.6675*P*] where *P* = (*F*_o_^2^ + 2*F*_c_^2^)/3
5394 reflections	(Δ/σ)_max_ = 0.001
229 parameters	Δρ_max_ = 0.41 e Å^−^^3^
0 restraints	Δρ_min_ = −0.24 e Å^−^^3^

### Special details

**Table:**

Geometry. All esds (except the esd in the dihedral angle between two l.s. planes) are estimated using the full covariance matrix. The cell esds are taken into account individually in the estimation of esds in distances, angles and torsion angles; correlations between esds in cell parameters are only used when they are defined by crystal symmetry. An approximate (isotropic) treatment of cell esds is used for estimating esds involving l.s. planes.

### Fractional atomic coordinates and isotropic or equivalent isotropic displacement parameters (Å^2^)

**Table:**

	*x*	*y*	*z*	*U*_iso_*/*U*_eq_	
C1	0.31969 (7)	0.48305 (5)	0.61877 (11)	0.01242 (16)	
C2	0.39767 (7)	0.54031 (5)	0.66026 (11)	0.01353 (17)	
C3	0.42548 (8)	0.58054 (5)	0.53374 (11)	0.01474 (17)	
H3	0.477724	0.619848	0.562029	0.018*	
C4	0.37653 (8)	0.56300 (5)	0.36575 (11)	0.01354 (17)	
C5	0.29811 (7)	0.50652 (5)	0.32350 (11)	0.01337 (17)	
H5	0.263797	0.495178	0.208904	0.016*	
C6	0.27031 (7)	0.46674 (5)	0.45055 (11)	0.01225 (16)	
C7	0.40926 (8)	0.60579 (5)	0.22916 (12)	0.01611 (18)	
H7A	0.490201	0.616399	0.266379	0.019*	
H7B	0.394974	0.574881	0.126786	0.019*	
C8	0.34694 (10)	0.67679 (6)	0.18696 (15)	0.0254 (2)	
H8	0.351201	0.710939	0.275650	0.031*	
C9	0.28622 (12)	0.69551 (9)	0.03483 (19)	0.0417 (3)	
H9A	0.280058	0.662727	−0.056904	0.050*	
H9B	0.248804	0.741721	0.017374	0.050*	
C11	0.14573 (8)	0.38762 (5)	0.25820 (11)	0.01383 (17)	
C12	0.19333 (7)	0.32850 (5)	0.19326 (11)	0.01273 (17)	
C13	0.13745 (8)	0.29999 (5)	0.03591 (11)	0.01344 (17)	
H13	0.168944	0.259885	−0.009373	0.016*	
C14	0.03563 (8)	0.32995 (5)	−0.05550 (11)	0.01455 (17)	
C15	−0.00827 (8)	0.39029 (5)	0.00937 (12)	0.01768 (18)	
H15	−0.076237	0.411947	−0.053674	0.021*	
C16	0.04695 (8)	0.41898 (5)	0.16599 (12)	0.01738 (18)	
H16	0.016701	0.460156	0.209547	0.021*	
C17	−0.02679 (8)	0.29803 (5)	−0.22437 (12)	0.01738 (18)	
H17A	0.000598	0.247391	−0.234350	0.021*	
H17B	−0.107102	0.294761	−0.230608	0.021*	
C18	−0.01256 (9)	0.34388 (6)	−0.36835 (12)	0.01971 (19)	
H18	0.060227	0.347660	−0.383003	0.024*	
C19	−0.09401 (11)	0.37929 (7)	−0.47598 (15)	0.0310 (3)	
H19A	−0.167876	0.376757	−0.465183	0.037*	
H19B	−0.078789	0.407319	−0.564243	0.037*	
C20	0.21477 (9)	0.47287 (6)	0.81429 (13)	0.0207 (2)	
H20A	0.240600	0.521609	0.860138	0.031*	
H20B	0.203875	0.441120	0.904430	0.031*	
H20C	0.144028	0.478054	0.726983	0.031*	
C21	0.52459 (8)	0.60736 (6)	0.87749 (12)	0.01920 (19)	
H21A	0.583203	0.600002	0.820618	0.029*	
H21B	0.557367	0.605531	0.999413	0.029*	
H21C	0.489348	0.655567	0.846148	0.029*	
C22	0.33781 (9)	0.23855 (6)	0.23543 (13)	0.0216 (2)	
H22A	0.286150	0.196974	0.225908	0.032*	
H22B	0.409272	0.226083	0.315373	0.032*	
H22C	0.349503	0.248886	0.125186	0.032*	
O1	0.19375 (6)	0.41001 (4)	0.42283 (8)	0.01578 (14)	
O2	0.29616 (6)	0.44052 (4)	0.74330 (8)	0.01493 (14)	
O3	0.44286 (6)	0.55060 (4)	0.82841 (8)	0.01778 (15)	
O4	0.29183 (6)	0.30235 (4)	0.29352 (8)	0.01611 (14)	

### Atomic displacement parameters (Å^2^)

**Table:**

	*U*^11^	*U*^22^	*U*^33^	*U*^12^	*U*^13^	*U*^23^
C1	0.0147 (4)	0.0123 (4)	0.0112 (4)	0.0010 (3)	0.0052 (3)	0.0016 (3)
C2	0.0140 (4)	0.0152 (4)	0.0111 (4)	0.0007 (3)	0.0031 (3)	−0.0005 (3)
C3	0.0159 (4)	0.0144 (4)	0.0142 (4)	−0.0019 (3)	0.0046 (3)	0.0003 (3)
C4	0.0148 (4)	0.0140 (4)	0.0130 (4)	0.0015 (3)	0.0059 (3)	0.0018 (3)
C5	0.0152 (4)	0.0148 (4)	0.0106 (4)	0.0008 (3)	0.0044 (3)	−0.0003 (3)
C6	0.0132 (4)	0.0108 (4)	0.0134 (4)	−0.0001 (3)	0.0048 (3)	−0.0011 (3)
C7	0.0185 (4)	0.0170 (4)	0.0148 (4)	−0.0007 (3)	0.0078 (3)	0.0023 (3)
C8	0.0328 (6)	0.0199 (5)	0.0300 (5)	0.0048 (4)	0.0193 (5)	0.0087 (4)
C9	0.0348 (7)	0.0478 (8)	0.0461 (8)	0.0139 (6)	0.0169 (6)	0.0296 (6)
C11	0.0170 (4)	0.0135 (4)	0.0117 (4)	−0.0037 (3)	0.0053 (3)	−0.0020 (3)
C12	0.0136 (4)	0.0122 (4)	0.0128 (4)	−0.0012 (3)	0.0042 (3)	0.0017 (3)
C13	0.0160 (4)	0.0122 (4)	0.0133 (4)	−0.0012 (3)	0.0060 (3)	−0.0004 (3)
C14	0.0157 (4)	0.0150 (4)	0.0131 (4)	−0.0024 (3)	0.0042 (3)	0.0000 (3)
C15	0.0158 (4)	0.0184 (4)	0.0180 (4)	0.0014 (3)	0.0032 (3)	0.0000 (3)
C16	0.0186 (4)	0.0154 (4)	0.0192 (4)	0.0012 (3)	0.0069 (4)	−0.0027 (3)
C17	0.0183 (4)	0.0190 (4)	0.0138 (4)	−0.0020 (3)	0.0027 (3)	−0.0019 (3)
C18	0.0239 (5)	0.0202 (5)	0.0162 (4)	0.0025 (4)	0.0074 (4)	−0.0011 (3)
C19	0.0387 (7)	0.0305 (6)	0.0229 (5)	0.0107 (5)	0.0069 (5)	0.0061 (4)
C20	0.0256 (5)	0.0204 (5)	0.0212 (5)	−0.0012 (4)	0.0149 (4)	0.0001 (4)
C21	0.0182 (4)	0.0236 (5)	0.0157 (4)	−0.0072 (4)	0.0046 (3)	−0.0041 (4)
C22	0.0222 (5)	0.0168 (4)	0.0241 (5)	0.0055 (4)	0.0034 (4)	−0.0007 (4)
O1	0.0210 (3)	0.0155 (3)	0.0116 (3)	−0.0062 (2)	0.0057 (2)	−0.0028 (2)
O2	0.0198 (3)	0.0139 (3)	0.0127 (3)	0.0008 (2)	0.0072 (2)	0.0030 (2)
O3	0.0199 (3)	0.0222 (3)	0.0105 (3)	−0.0071 (3)	0.0027 (3)	−0.0013 (2)
O4	0.0162 (3)	0.0158 (3)	0.0150 (3)	0.0026 (2)	0.0020 (2)	−0.0002 (2)

### Geometric parameters (Å, º)

**Table:**

C1—O2	1.3804 (10)	C13—H13	0.9500
C1—C6	1.3911 (12)	C14—C15	1.3938 (13)
C1—C2	1.3987 (12)	C14—C17	1.5158 (13)
C2—O3	1.3635 (11)	C15—C16	1.3913 (13)
C2—C3	1.3945 (12)	C15—H15	0.9500
C3—C4	1.3934 (12)	C16—H16	0.9500
C3—H3	0.9500	C17—C18	1.5017 (14)
C4—C5	1.3919 (13)	C17—H17A	0.9900
C4—C7	1.5151 (12)	C17—H17B	0.9900
C5—C6	1.3938 (12)	C18—C19	1.3197 (15)
C5—H5	0.9500	C18—H18	0.9500
C6—O1	1.3783 (11)	C19—H19A	0.9500
C7—C8	1.4937 (14)	C19—H19B	0.9500
C7—H7A	0.9900	C20—O2	1.4292 (12)
C7—H7B	0.9900	C20—H20A	0.9800
C8—C9	1.3235 (18)	C20—H20B	0.9800
C8—H8	0.9500	C20—H20C	0.9800
C9—H9A	0.9500	C21—O3	1.4263 (11)
C9—H9B	0.9500	C21—H21A	0.9800
C11—C16	1.3813 (13)	C21—H21B	0.9800
C11—O1	1.3902 (11)	C21—H21C	0.9800
C11—C12	1.4005 (13)	C22—O4	1.4301 (12)
C12—O4	1.3652 (11)	C22—H22A	0.9800
C12—C13	1.3969 (12)	C22—H22B	0.9800
C13—C14	1.3975 (13)	C22—H22C	0.9800
			
O2—C1—C6	120.12 (8)	C13—C14—C17	120.83 (8)
O2—C1—C2	120.38 (8)	C16—C15—C14	120.33 (9)
C6—C1—C2	119.41 (8)	C16—C15—H15	119.8
O3—C2—C3	125.18 (8)	C14—C15—H15	119.8
O3—C2—C1	114.68 (8)	C11—C16—C15	120.04 (9)
C3—C2—C1	120.12 (8)	C11—C16—H16	120.0
C4—C3—C2	119.89 (8)	C15—C16—H16	120.0
C4—C3—H3	120.1	C18—C17—C14	112.21 (8)
C2—C3—H3	120.1	C18—C17—H17A	109.2
C5—C4—C3	120.30 (8)	C14—C17—H17A	109.2
C5—C4—C7	120.15 (8)	C18—C17—H17B	109.2
C3—C4—C7	119.55 (8)	C14—C17—H17B	109.2
C4—C5—C6	119.52 (8)	H17A—C17—H17B	107.9
C4—C5—H5	120.2	C19—C18—C17	124.63 (10)
C6—C5—H5	120.2	C19—C18—H18	117.7
O1—C6—C1	114.97 (8)	C17—C18—H18	117.7
O1—C6—C5	124.29 (8)	C18—C19—H19A	120.0
C1—C6—C5	120.75 (8)	C18—C19—H19B	120.0
C8—C7—C4	112.72 (8)	H19A—C19—H19B	120.0
C8—C7—H7A	109.0	O2—C20—H20A	109.5
C4—C7—H7A	109.0	O2—C20—H20B	109.5
C8—C7—H7B	109.0	H20A—C20—H20B	109.5
C4—C7—H7B	109.0	O2—C20—H20C	109.5
H7A—C7—H7B	107.8	H20A—C20—H20C	109.5
C9—C8—C7	124.67 (12)	H20B—C20—H20C	109.5
C9—C8—H8	117.7	O3—C21—H21A	109.5
C7—C8—H8	117.7	O3—C21—H21B	109.5
C8—C9—H9A	120.0	H21A—C21—H21B	109.5
C8—C9—H9B	120.0	O3—C21—H21C	109.5
H9A—C9—H9B	120.0	H21A—C21—H21C	109.5
C16—C11—O1	120.11 (8)	H21B—C21—H21C	109.5
C16—C11—C12	120.65 (8)	O4—C22—H22A	109.5
O1—C11—C12	118.99 (8)	O4—C22—H22B	109.5
O4—C12—C13	125.09 (8)	H22A—C22—H22B	109.5
O4—C12—C11	115.92 (8)	O4—C22—H22C	109.5
C13—C12—C11	118.97 (8)	H22A—C22—H22C	109.5
C12—C13—C14	120.59 (8)	H22B—C22—H22C	109.5
C12—C13—H13	119.7	C6—O1—C11	118.29 (7)
C14—C13—H13	119.7	C1—O2—C20	113.39 (7)
C15—C14—C13	119.35 (8)	C2—O3—C21	116.94 (7)
C15—C14—C17	119.82 (8)	C12—O4—C22	116.91 (7)
			
O2—C1—C2—O3	−1.95 (12)	O4—C12—C13—C14	178.72 (8)
C6—C1—C2—O3	−178.56 (8)	C11—C12—C13—C14	0.02 (13)
O2—C1—C2—C3	176.58 (8)	C12—C13—C14—C15	2.04 (14)
C6—C1—C2—C3	−0.03 (13)	C12—C13—C14—C17	−178.48 (8)
O3—C2—C3—C4	177.54 (9)	C13—C14—C15—C16	−2.02 (14)
C1—C2—C3—C4	−0.83 (14)	C17—C14—C15—C16	178.49 (9)
C2—C3—C4—C5	1.41 (14)	O1—C11—C16—C15	−171.93 (9)
C2—C3—C4—C7	−178.55 (8)	C12—C11—C16—C15	2.18 (14)
C3—C4—C5—C6	−1.12 (13)	C14—C15—C16—C11	−0.08 (15)
C7—C4—C5—C6	178.84 (8)	C15—C14—C17—C18	77.59 (11)
O2—C1—C6—O1	4.08 (12)	C13—C14—C17—C18	−101.89 (10)
C2—C1—C6—O1	−179.29 (8)	C14—C17—C18—C19	−115.54 (12)
O2—C1—C6—C5	−176.30 (8)	C1—C6—O1—C11	−176.28 (8)
C2—C1—C6—C5	0.32 (13)	C5—C6—O1—C11	4.12 (13)
C4—C5—C6—O1	179.83 (8)	C16—C11—O1—C6	−91.49 (11)
C4—C5—C6—C1	0.25 (13)	C12—C11—O1—C6	94.29 (10)
C5—C4—C7—C8	96.87 (11)	C6—C1—O2—C20	−99.35 (10)
C3—C4—C7—C8	−83.17 (11)	C2—C1—O2—C20	84.06 (10)
C4—C7—C8—C9	−123.62 (12)	C3—C2—O3—C21	0.31 (14)
C16—C11—C12—O4	179.03 (8)	C1—C2—O3—C21	178.76 (8)
O1—C11—C12—O4	−6.79 (12)	C13—C12—O4—C22	−3.25 (13)
C16—C11—C12—C13	−2.15 (13)	C11—C12—O4—C22	175.49 (8)
O1—C11—C12—C13	172.03 (8)		

### Hydrogen-bond geometry (Å, º)

**Table:**

*D*—H···*A*	*D*—H	H···*A*	*D*···*A*	*D*—H···*A*
C20—H20*A*···O3	0.98	2.66	3.1461 (13)	111
C21—H21*B*···O2^i^	0.98	2.54	3.4292 (12)	151
C22—H22*A*···O2^ii^	0.98	2.50	3.2885 (12)	138

Symmetry codes: (i) −*x*+1, −*y*+1, −*z*+2; (ii) *x*, −*y*+1/2, *z*−1/2.
